# Extracellular HMGB1 Impairs Macrophage-Mediated Efferocytosis by Suppressing the Rab43-Controlled Cell Surface Transport of CD91

**DOI:** 10.3389/fimmu.2022.767630

**Published:** 2022-03-22

**Authors:** Yao Wang, Wen Zhang, Yu Xu, Di Wu, Zhan Gao, Jianchun Zhou, Hang Qian, Binfeng He, Guansong Wang

**Affiliations:** ^1^ Institute of Respiratory Diseases, Department of Pulmonary and Critical Care Medicine, Xinqiao Hospital, Third Military Medical University, Chongqing, China; ^2^ Department of Respiratory and Critical Care Medicine, The First Affiliated Hospital of Chongqing Medical University, Chongqing, China; ^3^ Department of Pulmonary and Critical Care Medicine, Zhongshan Hospital, Fudan University, Shanghai, China

**Keywords:** Rab43, macrophage, HMGB1, ARDS, CD91

## Abstract

High-mobility group box 1 (HMGB1) protein can impair phagocyte function by suppressing the macrophage-mediated clearance of apoptotic cells (ACs), thereby delaying inflammation resolution in the lungs and allowing the progression of acute lung injury (ALI) and acute respiratory distress syndrome (ARDS). However, the precise mechanism underlying this HMGB1-mediated inhibition of efferocytosis remains unknown. The aim of this study was to determine the effect of HMGB1 on macrophage-mediated efferocytosis. We discovered that HMGB1 prevented efferocytosis by bone marrow-derived macrophages (BMDMs) and suppressed the expression of Ras-related GTP-binding protein 43 (Rab43), a member of the Ras-associated binding (Rab) family. The downregulation of Rab43 expression resulted in impaired clearance of apoptotic thymocytes by BMDMs. Subsequent analysis of HMGB1-treated and Rab43-deficient BMDMs revealed the inhibited transport of cluster of differentiation 91 (CD91), a phagocyte recognition receptor, from the cytoplasm to the cell surface. Notably, Rab43 directly interacted with CD91 to mediate its intercellular trafficking. Furthermore, Rab43 knockout delayed the inflammation resolution and aggravated the lung tissue damage in mice with ALI. Therefore, our results provide evidence that HMGB1 impairs macrophage-mediated efferocytosis and delays inflammation resolution by suppressing the Rab43-regulated anterograde transport of CD91, suggesting that the restoration of Rab43 levels is a promising strategy for attenuating ALI and ARDS in humans.

## Introduction

Acute lung injury (ALI) and acute respiratory distress syndrome (ARDS) are diseases with multiple complications that can seriously threaten the life and quality of living of a patient (1). These conditions can be caused by a variety of pulmonary (e.g., pneumonia, aspiration) or ex-pulmonary (e.g., sepsis, pancreatitis, trauma) insults, leading to the development of nonhydrostatic pulmonary edema (2). Hence, excessive and uncontrolled inflammatory responses in the lungs are integral to the occurrence and development of ARDS.

During ALI or ARDS pathogenesis, multiple inflammatory mediators are released from the damaged lung tissue. These mediators impact the lung tissue microenvironment and may determine the outcome in ARDS patients (3). One example is high-mobility group box 1 (HMGB1), a highly conserved, ubiquitous DNA-binding nuclear protein, which plays a key role in the initiation of innate and adaptive immune responses (4, 5). As a late-acting mediator, HMGB1 can be passively released by almost all cell types (6). In mice, inhibition of HMGB1 activity *via* antibody targeting revealed a protective effect against lipopolysaccharide (LPS) lethality, whereas increasing HMGB1 activity resulted in worsened endotoxemia and LPS lethality (7). Further studies demonstrated that HMGB1 contributed to endotoxin-induced ALI by activating the nuclear translocation of nuclear factor (NF)-κB and increasing the levels of proinflammatory cytokines and adhesion molecules (8). However, extracellular HMGB1 can also inhibit the function of macrophages, thereby preventing the clearance of apoptotic cells (9, 10).

Macrophage phagocytic function is typically associated with the engulfment of dying cells, pathogens, and foreign particulates to maintain lung homeostasis (11, 12). A study with the ALI mouse model showed that one of the major functions of macrophages is to engulf apoptotic neutrophils to induce the resolution of lung inflammation and lung tissue injury (13). Macrophage dysfunction results in the delayed clearing of apoptotic neutrophils, resulting in their excessive accumulation in the alveoli and distal bronchioles, exacerbating the inflammatory response and histopathological damage in the lungs (14). For example, Grégoire et al. (15) discovered that the macrophage engulfment of neutrophil extracellular traps and apoptotic neutrophils was suppressed in ARDS patients. However, the precise mechanism underlying the impaired macrophage-mediated efferocytosis in ALI and ARDS is still unknown.

The recognition and binding of apoptotic cells by macrophage surface receptors are essential for normal macrophage efferocytosis. As a member of the low-density lipoprotein receptor (LDLR) superfamily, cluster of differentiation 91 (CD91), also known as LDL receptor-related protein 1 (LRP1), can bind to apoptotic cells and initiate phagocytosis (16–19). Receptors such as CD91 are known to participate in the whole process of pulmonary inflammation, from occurrence to regression (20). In addition, CD91 deficiency was found to delay the clearance of apoptotic cells and contribute to the high mortality rates in LPS-treated mice (21, 22). Thus, CD91 is a critical receptor that facilitates the innate immune responses and phagocytosis in macrophages.

Ras-associated binding (Rab) GTPases form the largest branch of the Ras-related small GTPase superfamily, which can precisely regulate the intracellular trafficking of receptors, namely, the movement of newly synthesized receptors from the endoplasmic reticulum (ER) to the cell surface, endocytosis of receptor–ligand complexes from the cell surface, and translocation of the complexes to the endosomes (23). Early studies demonstrated that Rab GTPases are involved in regulating macrophage phagocytic receptor expression (24), post-phagocytosis processing, and downstream signal transduction (25). Rab43, a member of the Rab family, is reportedly involved in transporting G protein-coupled receptors (GPCRs) (26) and regulating downstream signal transduction (27). However, little is known regarding the function of Rab43 on macrophage activity.

Thus, the aim of this study was to investigate the role of HMGB1 and determine the mechanism underlying the HMGB1-induced impaired efferocytosis in the LPS-induced ALI mouse model. Given that efferocytosis by macrophages was suppressed in ALI mouse models and ARDS patients (15, 28), we hypothesized that bronchoalveolar lavage fluid (BALF) can impair the macrophage-mediated engulfment of apoptotic cells. Therefore, we treated mouse bone marrow-derived macrophages (BMDMs) with BALF from the ALI mouse model and recombinant HMGB1 (rHMGB1) to evaluate the effect on apoptotic cells. Previous studies reported that Rab43 participates in the phagocytosis of *Mycobacterium tuberculosis* and *Staphylococcus aureus* (29). Hence, we also evaluated the role of Rab43 in macrophage-mediated efferocytosis. We generated myeloid cell-specific *Rab43*-knockout mice (*Rab43^floxp/floxp^/LysMCre^+/+^* mice; hereafter referred to as Rab43-cKO mice), as previously described (30) and BMDMs were extracted from Rab43-cKO and *Rab43^floxp/floxp^/LysMCre^−/−^* (Rab43-C, as the wild-type control) mice. Our findings may provide novel insights regarding the HMGB1-mediated inhibition of apoptotic cell clearance and identify a potential therapeutic approach for the treatment of ALI and ARDS in humans.

## Results

### Extracellular HMGB1 Impairs Macrophage-Mediated Efferocytosis

**Figure 1:**
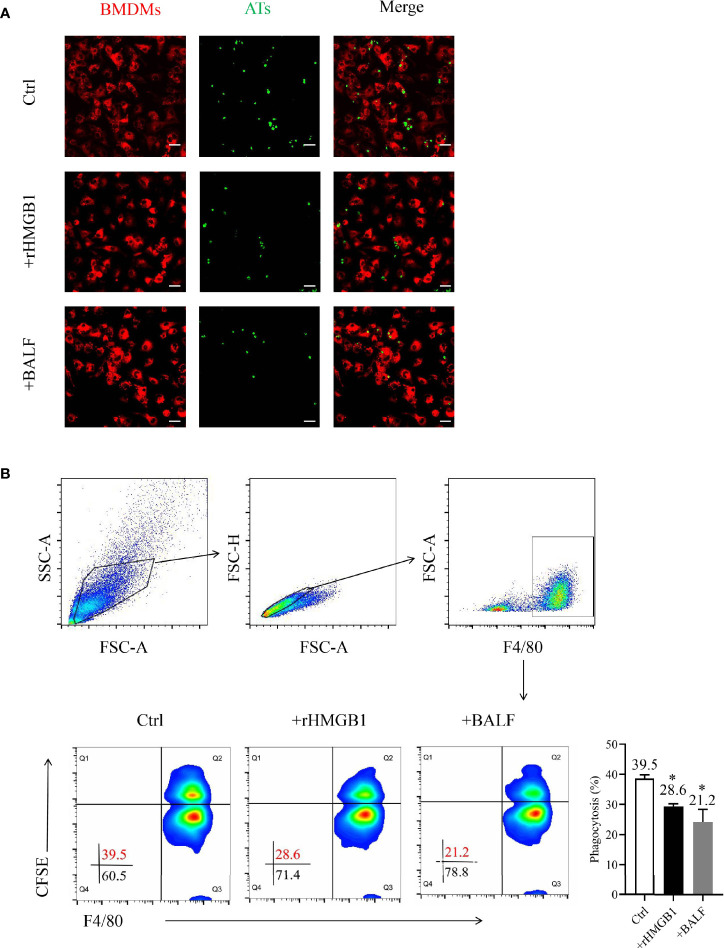
Effects of recombinant HMGB1 (rHMGB1) treatment on macrophage-mediated efferocytosis. **(A)** Analysis of macrophage-mediated engulfment of apoptotic thymocytes (ATs) by confocal microscopy. The bone marrow-derived macrophages (BMDMs) were preincubated for 12 h with 2 μg/ml rHMGB1 or bronchoalveolar lavage fluid (BALF) extracted from mice with acute lung injury (ALI). The CFSE-labeled ATs were co-cultured with Dil-labeled BMDMs for 2 h. The free and attached ATs were removed by extensive washing with cold phosphate buffered saline (PBS). Representative fluorescence images of macrophage efferocytosis are shown in the left panel, and the numbers of ATs are shown in a bar graph in the right panel. Scale bars = 20.0 μm. **(B)** Analysis of the macrophage-mediated efferocytosis through flow cytometry. The F4/80-labeled BMDMs were pretreated as mentioned above and co-cultured with CFSE-labeled ATs (1:10) for 2 h. Representative images of the macrophages are shown in the left panel, and the phagocytosis rates are presented in the right panel. *P < 0.05 versus the wild-type (WT) group.

Confocal microscopy and flow cytometry showed that macrophage-mediated efferocytosis was suppressed in BMDMs after treatment with BALF (**Figures 1A, B**). This finding corroborated previous reports showing that the BALF contains harmful metabolites and that extracellular HMGB1 inhibits macrophage activity during efferocytosis (31, 32). In addition, the HMGB1 levels in the BALF increased with prolonged LPS exposure (**Figure S1A**). Notably, the expression level of HMGB1 in the BALF reached a high level at 24 h; this finding was consistent with the result of a previous study in a sepsis-induced ARDS model (33).

To determine the function of HMGB1 on macrophage engulfment, we evaluated the efferocytosis by BMDMs after treatment with rHMGB1 for 12 h. The phagocytosis index (PI) of the treatment group decreased to 21% when compared with that of the control group of approximately 40% PI (**Figure 1B**). Moreover, treatment of BMDMs with anti-HMGB1 neutralizing antibody significantly attenuated the suppressive effect of BALF on phagocytosis (**Figure S1B**). Collectively, these data suggested that HMGB1-treated BMDMs displayed a lower phagocytosis rate, implying that rHMGB1 treatment impaired macrophage-mediated efferocytosis.

### HMGB1 Suppressed the Expression of Rab43

**Figure 2:**
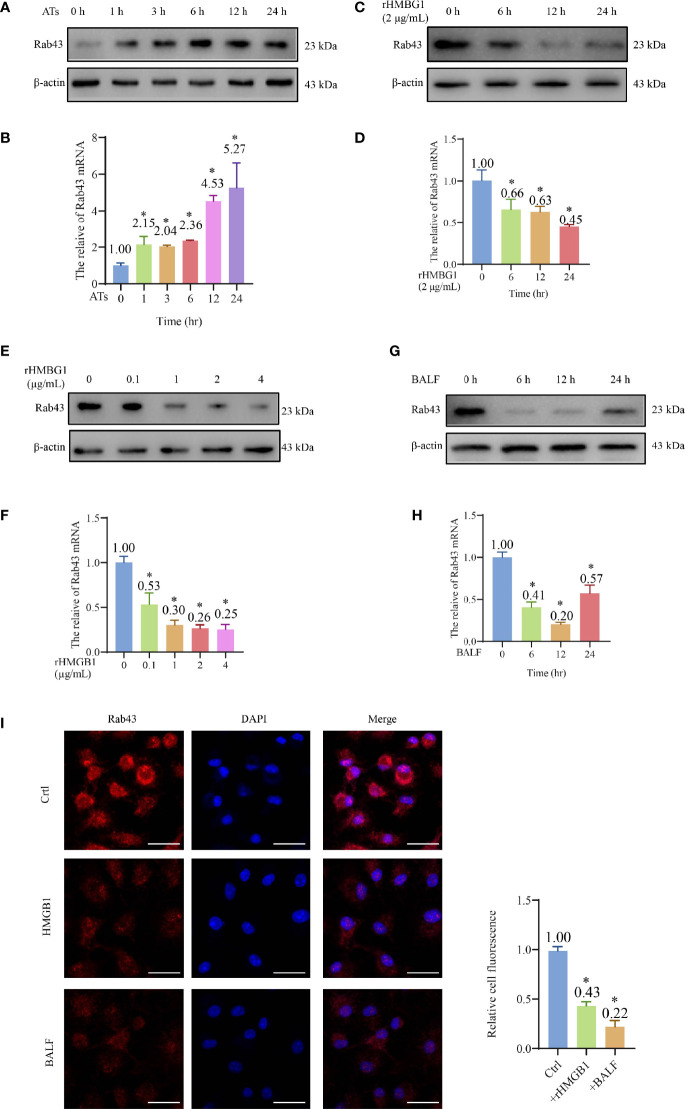
HMGB1 suppressed the expression of Rab43. **(A, B)** Western blot and qPCR analyses of Rab43 mRNA and protein expression levels. The bone marrow-derived macrophages (BMDMs) were co-cultured with ATs (1:10) at the indicated time points (0, 1, 3, 6, 12, and 24 h). *P < 0.05 versus 0 h. **(C–H)** Western blot and qPCR analyses of Rab43 mRNA and protein levels. The BMDMs were treated with 2 μg/ml recombinant HMGB1 (rHMGB1) at indicated time points (0, 6, 12, and 24 h) **(C, D)**. The BMDMs were pretreated with 0.1, 1, 2, and 4 μg/ml rHMGB1 for 12 h **(E, F)**. C57BL/6 mice were treated with 5 mg/kg LPS for 24 h *via* i.t. injection and the BMDMs were treated with bronchoalveolar lavage fluid (BALF) from the mice at indicated time points (0, 6, 12, and 24 h) **(G, H)**. *P < 0.05 versus 0 h. **(I)** The Rab43 protein expression in the BMDMs was determined after treatment with rHMGB1 and BALF for 12 h by immunofluorescence staining. The nuclei were stained with DAPI. Scale bars = 20 μm. *P < 0.05 versus the control group.

Western blotting showed that the protein expression of Rab43 of BMDMs reached high levels at 12 and 24 h after co-culture with apoptotic thymocytes (**Figures 2A** and **S2A**), indicating that Rab43 might be involved in efferocytosis by macrophages. The same results were observed at the mRNA level with quantitative polymerase chain reaction (qPCR) (**Figure 2B**).

After treatment of BMDMs with 2 μg/ml rHMGB1 to induce an inflammatory environment, the Rab43 expression level in the cells decreased compared with that of the control group (**Figures 2C, D** and **S2B**). Additionally, we treated the BMDMs with different doses of rHMGB1 for 12 h and observed that the Rab43 expression level decreased after 0.1, 1, 2, and 4 μg/ml rHMGB1 treatments (**Figures 2E, F** and **S2C**). A similar effect was observed when the BMDMs were treated with BALF. Specifically, the expression of Rab43 significantly decreased 6 h after the treatment and reached the lowest value at 12 h (**Figures 2G, H** and **S2D**). Furthermore, confocal laser-scanning microscopy (CLSM) observations confirmed that both rHMGB1 and ALI mouse BALF treatments resulted in the downregulated expression of Rab43 in the cytoplasm of BMDMs (**Figure 2I**).

We further explored the effect of HMGB1 on the regulation of other Rab GTPases (such as Rab7 and Rab11a), which also participate in phagocytosis (24, 34). After BMDMs were treated with HMGB1 for 24 h, the Rab11a expression level decreased by 60%, whereas there was no obvious change in the expression level of Rab7 (**Figure S2E**). These data suggested that HMGB1 impairs the macrophage phagocytosis of apoptotic thymocytes by suppressing Rab43 and other Rab GTPases.

### Rab43 Deficiency Impaired Efferocytosis by Macrophages

**Figure 3:**
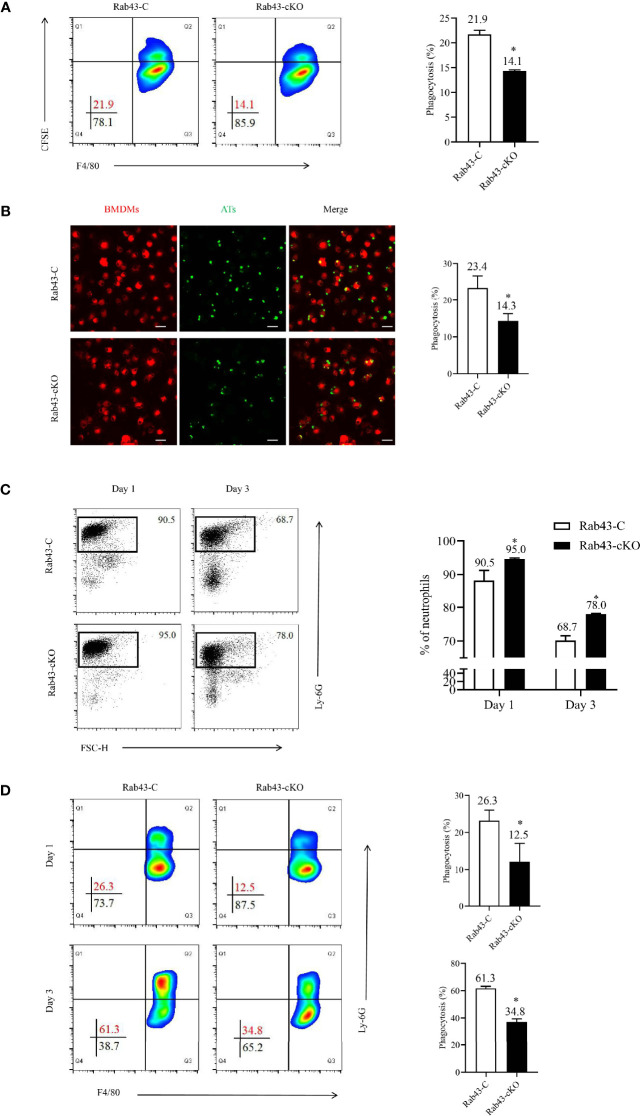
Rab43 deficiency impaired efferocytosis by macrophages. **(A, B)** The efferocytosis by Rab43-C and Rab43-cKO bone marrow-derived macrophages (BMDMs) *in vitro* analyzed by flow cytometry and CLSM. The CFSE-labeled apoptotic thymocytes (Ats) were co-cultured with BMDMs (10:1) for 2 h. The free and attached ATs were removed by extensive washing with cold phosphate-buffered saline (PBS). The engulfed cells were subsequently incubated with anti-F4/80 antibody or Dil Stain. *P < 0.05 versus the Rab43-C group. **(C)** The percentages of neutrophils in BALF samples detected using flow cytometry. The Rab43-C and Rab43-cKO mice were treated (i.t.) with 5 mg/kg lipopolysaccharide (LPS) for 1 and 3 days. The BALF samples were subsequently collected and incubated with anti-Ly6G antibody. **(D)** Macrophage-mediated efferocytosis *in vivo* was analyzed using flow cytometry. The BALF was collected from Rab43-C and Rab43-cKO mice after treatment with 5 mg/kg LPS (i.t.) for 1 and 3 days. The phagocytosis rate is expressed as the phagocytosis index (PI), which is the number of cells ingested (F4/80+Ly6G+) over the total number of macrophages (F4/80+). *P < 0.05 versus the Rab43-C group.

The PI in Rab43-cKO and Rab43-C BMDMs was 14.1 and 21.9%, respectively, suggesting that the deletion of *Rab43* resulted in the impaired engulfment of apoptotic thymocytes *in vitro* (**Figure 3A**). In addition, a lower number of apoptotic thymocytes were engulfed by the Rab43-cKO BMDMs than by the Rab43-C BMDMs (**Figure 3B**). Moreover, Rab7 expression appeared to be suppressed in Rab43-cKO BMDMs, but not Rab11a expression (**Figure S3**).

To confirm the mechanism of Rab43-regulated efferocytosis by macrophages during inflammation, we established the ALI/ARDS mouse model *via* intratracheal administration of low-dose LPS (5 mg/kg). On day 1, the proportions of neutrophils in the BALF samples remained at high levels, with approximately 90.5 and 95.0% neutrophils observed in Rab43-C and Rab43-cKO mice, respectively. On day 3, the neutrophil proportions in the Rab43-C and Rab43-cKO groups decreased to 68.7 and 78.0%, respectively. Notably, the neutrophil proportion was significantly lower in Rab43-C mice than in Rab43-cKO mice (**Figure 3C**). We subsequently analyzed the efferocytosis by macrophages under the same conditions and collected the BALF samples from mice with ALI after 1 and 3 days. The macrophage-mediated efferocytosis in the Rab43-cKO group was significantly reduced compared to the Rab43-C group (**Figure 3D**). Overall, these findings suggested that Rab43 controls macrophage-mediated efferocytosis both *in vitro* and *in vivo*.

### Extracellular HMGB1 Suppressed CD91 Localization to the Cell Surface

**Figure 4:**
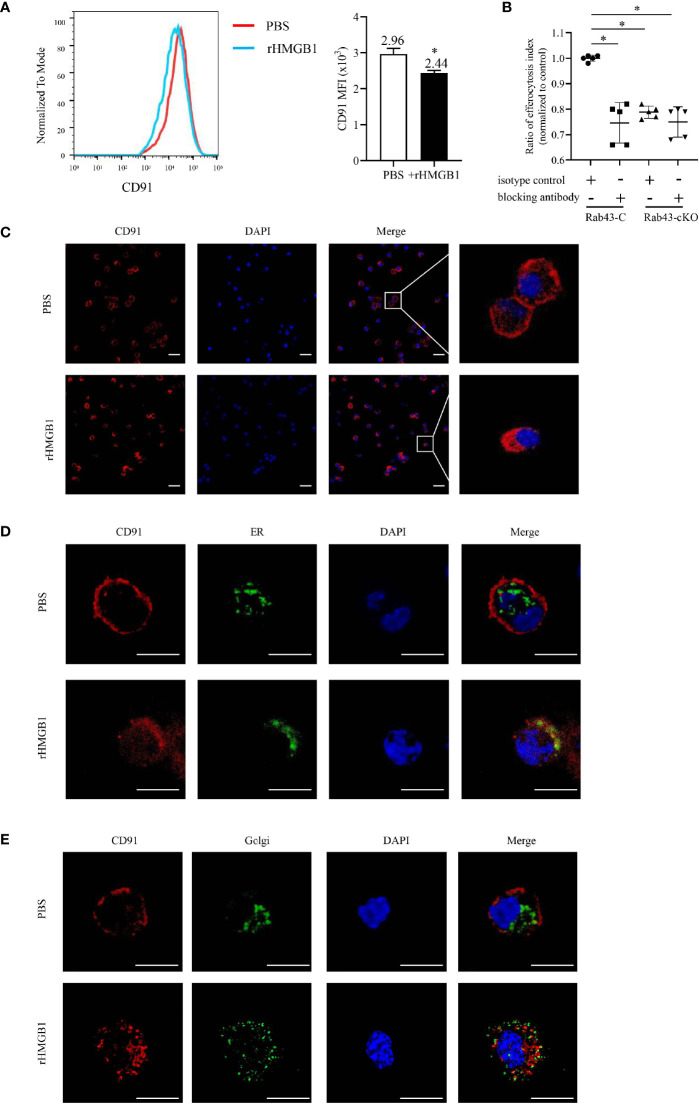
rHMGB1 suppressed CD91 expression on the cell surface. The bone marrow-derived macrophages (BMDMs) were treated with 2 μg/ml rHMGB1 for 12 h. **(A)** The CD91 level on the cell surface was detected using flow cytometry. *P < 0.05 versus the phosphate-buffered saline (PBS)-treated group. **(B)** Rab43-C or Rab43-cKO BMDMs were incubated with CD91-blocking antibody or IgG for 1 h, and then engulfment of apoptotic cells was measured by flow cytometry. *P < 0.05 versus the Rab43-C/IgG group. **(C)** Confocal laser-scanning microscopy (CLSM) to determine the subcellular localization of CD91. The BMDMs were stained with the anti-CD91 antibody. Red, CD91; blue, DAPI. Scale bars = 10.0 μm. **(D)** Co-localization of endogenous CD91 with the ER marker Calnexin. The BMDMs cultured in glass-bottom dishes were stained with anti-CD91 antibody and anti-Calnexin antibodies. Red, CD91; green, Calnexin; blue, DAPI. Scale bars = 10.0 μm. **(E)** Co-localization of endogenous CD91 with the Golgi body. The BMDMs were fixed with 4% formaldehyde solution for 10 min. A sufficient volume of Dual Detection Reagent was added to cover the monolayer cells (1:100 dilution). After blocking for 1 h, the cells were stained with anti-CD91 antibody. Red, CD91; green, Golgi body; blue, DAPI. Scale bars = 10.0 μm.

Various cell surface receptors are required for macrophage-mediated efferocytosis to facilitate the recognition, binding, and engulfment of apoptotic cells (35). Since HMGB1 suppressed the BMDM-mediated engulfment of apoptotic thymocytes, we hypothesized that the potential mechanism involved may be the regulation of receptor expression and function on the cell surfaces. Since CD91 was previously reported to be responsible for engulfing apoptotic cells *via* calreticulin binding (17, 36), we evaluated the effect of rHMGB1 treatment on CD91 expression. The CD91 levels significantly decreased in rHMGB1-treated BMDMs compared with those of the control cells (**Figure 4A**). The PI also differed between the Rab43-C/isotype-treated BMDM and the Rab43-C/CD91-blocking antibody-treated BMDM groups (**Figure 4B**). Subcellular distribution analysis also revealed that CD91 protein in phosphate-buffered saline (PBS)-treated BMDMs mainly localized on the cell membrane, whereas that in rHMGB1-treated BMDMs was distributed in the cytoplasm (**Figure 4C**). Further analysis showed that CD91 protein mainly co-localized with the endoplasmic reticulum (ER) marker rather than with the Golgi body marker in rHMGB1-treated BMDMs. In contrast, CD91 only partially co-localized with the ER marker (**Figure 4D**) and Golgi marker (**Figure 4E**) in PBS-treated cells. These results suggested that rHMGB1 treatment inhibited the transport of CD91 from the cytoplasm to the cell surface.

### *Rab43* Knockout Inhibits the Cell Surface Transport of Endogenous CD91

**Figure 5:**
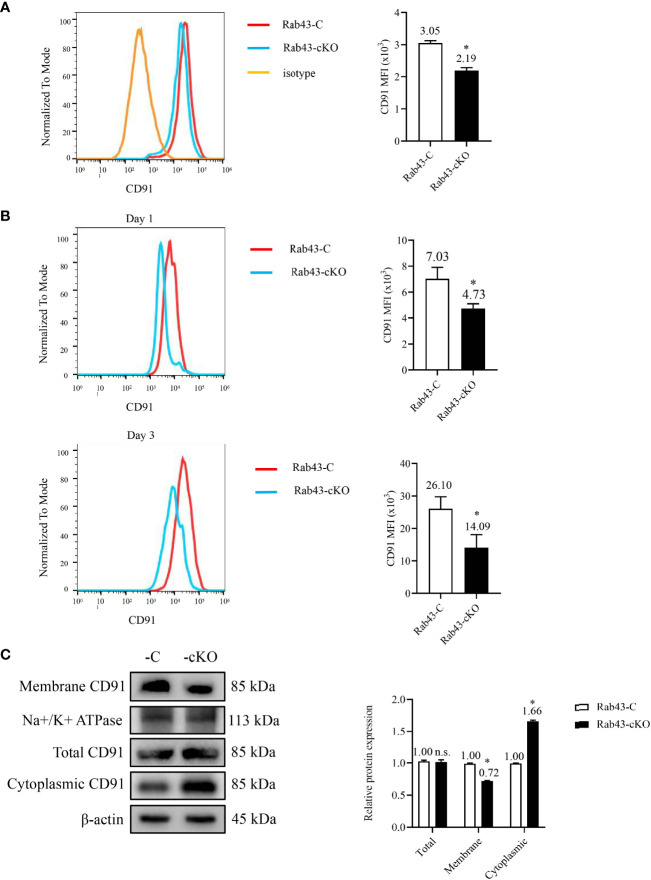
Effects of *Rab43* knockout on the cell surface transport of endogenous CD91. **(A)** The CD91 proteins on the cell surface were analyzed using flow cytometry. The bone marrow-derived macrophages (BMDMs) were isolated from Rab43-C and Rab43-cKO mice. *P < 0.05 versus the Rab43-C group. **(B)** The CD91 levels on the cell surface of BALF samples. The Rab43-C and Rab43-cKO mice were treated with LPS for 1 (upper panel) and 3 days (lower panel). The macrophages (F4/80^+^CD11c^+^CD11b^−^) in the BALF samples were also analyzed. *P < 0.05 versus the Rab43-C group. **(C)** The expression of total, cytoplasmic, and membrane CD91 in Rab43-C and Rab43-cKO BMDMs measured using western blot analyses. *P < 0.05 versus Rab43-C group. ns, not statistically significant.

To determine the relationship between the rHMGB1-mediated inhibition of CD91 protein transport to the cell surface and Rab43 expression, the level of CD91 in Rab43-cKO BMDMs was evaluated by flow cytometry. Compared with that on the Rab43-C BMDMs, the CD91 level on the cell surface of Rab43-cKO BMDMs was significantly decreased (**Figure 5A**). To further confirm the effects of *Rab43* knockout on CD91 expression *in vivo*, we analyzed the CD91 levels on the cell surface of macrophages (F4/80^+^CD11b^+^CD11c^−^) extracted from the BALF samples of mice with ALI. The CD91 level in the Rab43-cKO group was significantly decreased at days 1 and 3 after knockout compared with that of the Rab43-C group (**Figure 5B**; gating strategies are illustrated in **Figure S4A**). Western blotting showed that the expression level of CD91 at the cell surface was significantly decreased and the expression level in the cytoplasm was significantly increased in Rab43-cKO BMDMs compared with that in wild-type Rab43-C BMDMs (**Figure 5C**). However, there were no significant differences in the levels of the cell surface receptors MerTK and CD36 between groups, which are responsible for the regulation of apoptotic cell phagocytosis by macrophages (35) (**Figure S4B**). Together, these findings suggested that Rab43 plays a critical role in the cell surface transport of endogenous CD91 both *in vitro* and *in vivo*.

### Rab43 Regulates the Cell Surface Transport and Subcellular Localization of CD91

**Figure 6:**
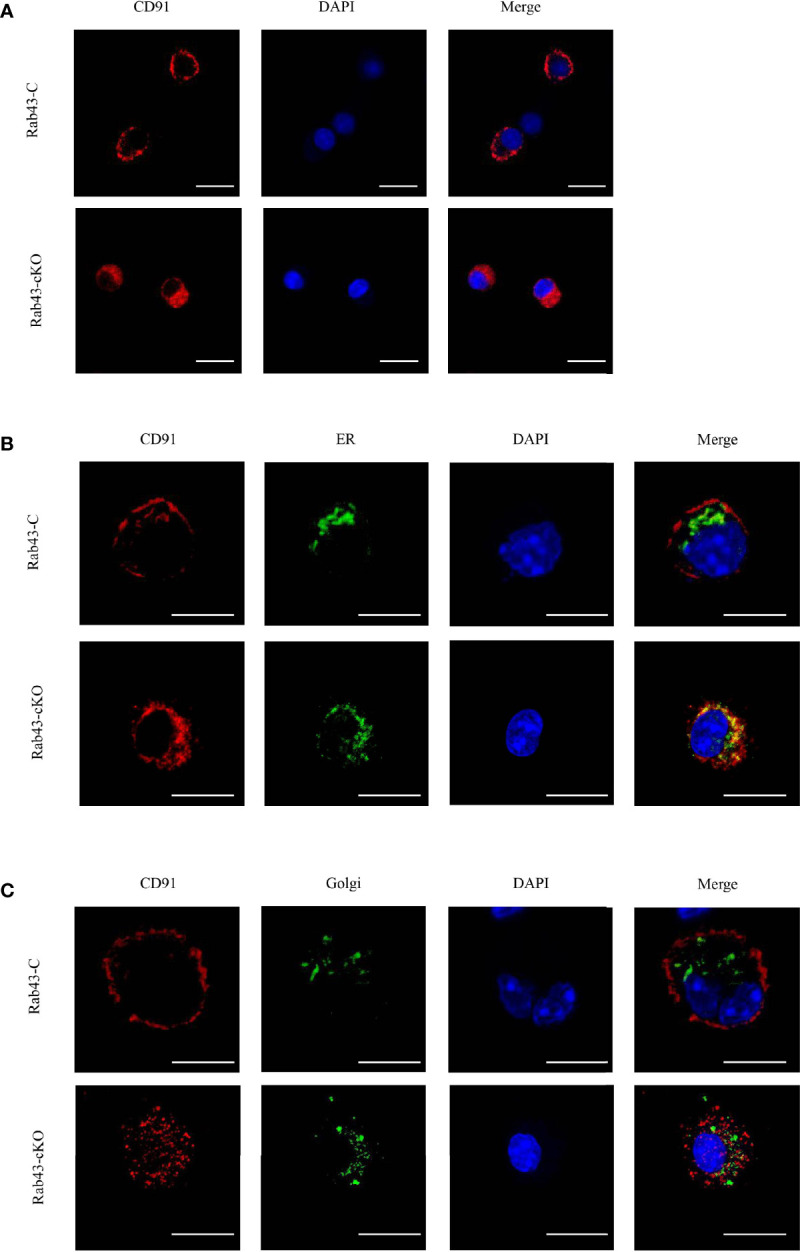
Rab43 regulates the transport of CD91 to the cell surface. **(A)** Localization of endogenous CD91 in Rab43-C and Rab43-cKO bone marrow-derived macrophages (BMDMs). The Rab43-C and Rab43-cKO BMDMs were stained with anti-CD91 antibody. Red, CD91; blue, DAPI. Scale bars = 10.0 μm. **(B)** Co-localization of endogenous CD91 with the ER marker Calnexin. The Rab43-C and Rab43-cKO BMDMs cultured in glass-bottom dishes were stained with anti-CD91 antibody and anti-Calnexin antibodies. Red, CD91; green, Calnexin; blue, DAPI. Scale bars = 10.0 μm. **(C)** Co-localization of endogenous CD91 with the Golgi body. The Rab43-C and Rab43-cKO BMDMs cultured in glass-bottom dishes were fixed with 4% formaldehyde solution for 10 min. Dual Detection Reagent was used to stain the Golgi body for 1 h. The cultured cells were then incubated with anti-CD91 antibody. Red, CD91; green, Golgi body; blue, DAPI. Scale bars = 10.0 μm.

**Figure 7:**
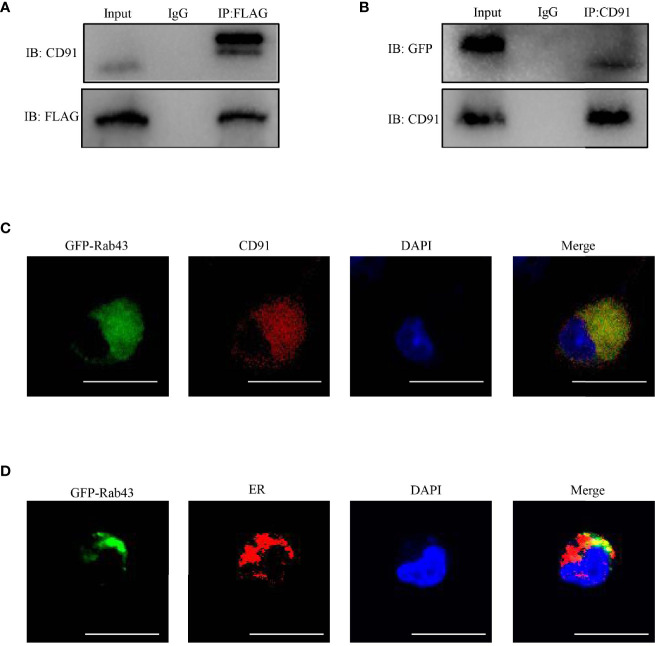
Rab43 directly interacts with CD91. **(A, B)** Co-immunoprecipitation of Rab43 with CD91. (Left panel) RAW264.7 cells were transfected with the pcDNA3.1-3xflag-Rab43 plasmid, and the cell lysates were co-immunoprecipitated with anti-FLAG antibody and then immunoblotted with anti-CD91 antibody. (Right panel) RAW264.7 cells were transfected with the pcDNA3.1-GFP-Rab43 plasmid, and the cell lysates were co-immunoprecipitated with anti-CD91 antibody and then immunoblotted with anti-GFP antibody. **(C, D)** RAW264.7 cells were transfected with plasmids expressing GFP-Rab43^WT^ and subsequently incubated with anti-CD91 antibody or anti-Calnexin antibodies. Green, GFP-Rab43-C; red, CD91 or Calnexin; blue, DAPI. Scale bars = 10.0 μm.

To further explore the function of Rab43 in the transport of CD91 to the cell surface, we analyzed the distribution of CD91 in Rab43-C and Rab43-cKO BMDMs using CLSM. The data revealed that endogenous CD91 was primarily localized on the cell surface of Rab43-C BMDMs, but was mainly distributed in the cytoplasm of Rab43-cKO BMDMs (**Figure 6A**). In addition, endogenous CD91 strongly co-localized with the ER of Rab43-cKO BMDMs (**Figure 6B**), whereas it minimally co-localized with the Golgi of Rab43-cKO BMDMs (**Figure 6C**), indicating that Rab43 is required for the normal transport of CD91 from the ER to the cell surface. Furthermore, we determined whether the interaction between Rab43 and CD91 was direct or indirect. The co-immunoprecipitation assay showed that immunoprecipitated CD91 was abundant in RAW264.7 cells expressing FLAG-Rab43 (**Figure 7A**). We also used CD91 to immunoprecipitate Rab43 in RAW264.7 cells expressing GFP-Rab43, which was also enriched (**Figure 7B**); CLSM demonstrated that the GFP-fused Rab43 proteins were predominantly distributed in the cytoplasm. After staining for endogenous CD91, we observed the strong co-localization of CD91 with GFP-Rab43 in the RAW264.7 cells (**Figure 7C**). Moreover, GFP-Rab43 was strongly co-localized with the ER (**Figure 7D**). Collectively, our findings suggest that Rab43 mediates the transport of CD91 from the ER to the cell membrane by directly interacting with CD91.

### *Rab43* Knockout Delays Inflammation Resolution and Exacerbates LPS-Induced ALI

**Figure 8:**
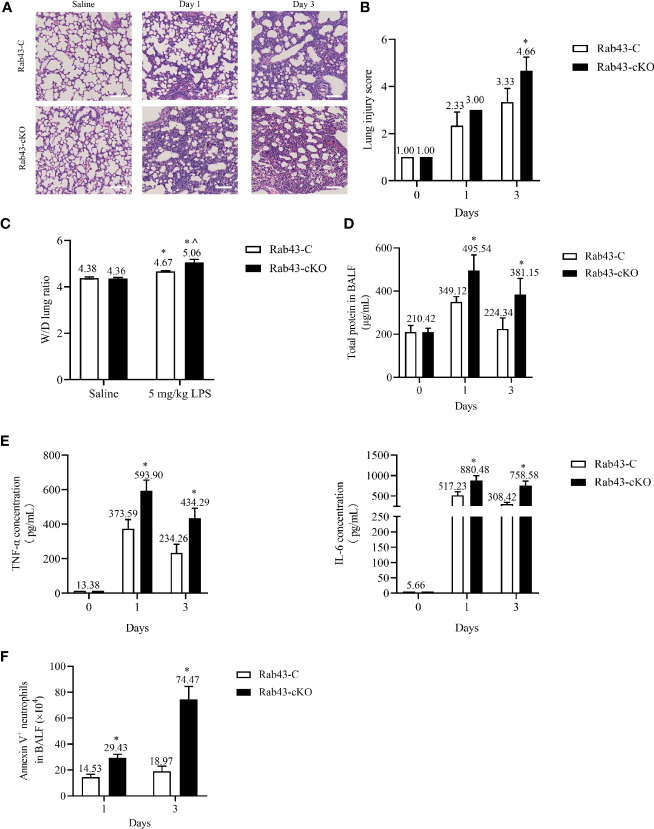
*Rab43* knockout delays inflammation resolution and exacerbates lipopolysaccharide (LPS)-induced acute lung injury (ALI) in mice. The Rab43-C and Rab43-cKO mice were treated with 5 mg/kg LPS (i.t.) at the indicated time points. **(A)** Hematoxylin and eosin (H&E) staining images showing the lung tissue injury. **(B)** The lung injury scores in the Rab43-C and Rab43-cKO groups. *P < 0.05 versus the Rab43-C group at indicated time points. **(C)** The level of lung edema was determined by measuring the wet-to-dry lung weight ratio. *P < 0.05 versus the Rab43-C saline group, ^*P* < 0.05, vs Rab43-C/5 mg/kg LPS group at indicated time points. **(D)** The protein concentrations in the bronchoalveolar lavage fluid (BALF) samples were analyzed using the bicinchoninic acid method. *P < 0.05 versus the Rab43-C group. **(E)** The levels of TNF-α (left panel) and IL-6 (right panel) in the BALF samples detected by enzyme-linked immunosorbent assay. *P < 0.05 versus the Rab43-C group at indicated time points. **(F)** Annexin V+/Ly6G+ cells were counted as apoptotic neutrophils using flow cytometry. *P < 0.05 versus the Rab43-C group at indicated time points.

To correlate the above-mentioned results with inflammation mediated-lung injury, ALI was induced in Rab43-C and Rab43-cKO mice. Histopathological analysis of the lung tissues revealed interstitial and intra-alveolar edema, diffuse alveolar damage, neutrophil infiltration, and lung tissue damage in the ALI mouse model (**Figures 8A, B**). The wet-to-dry (W/D) lung weight ratio (**Figure 8C**) and protein concentration (**Figure 8D**) in the Rab43-cKO group were significantly elevated compared with those in the Rab43-C group. Furthermore, the levels of inflammatory cytokines tumor necrosis factor (TNF)-α and interleukin (IL)-6 were elevated in both groups of mice after intratracheal treatment with 5 mg/kg LPS for 1 and 3 days (**Figure 8E**). Notably, the TNF-α and IL-6 levels were higher in the Rab43-cKO/LPS-treated group than in the Rab43-C/LPS-treated group. Moreover, the number of apoptotic neutrophils in the BALF of Rab43-cKO was higher than that in Rab43-C mice at days 1 and 3 after LPS administration (**Figure 8F**), suggesting that Rab43 deficiency impaired the efferocytosis. Taken together, these data suggest that the deletion of *Rab43* delays the inflammatory resolution and exacerbates the lung tissue damage in mice with ALI.

## Discussion

The findings of this study revealed that HMGB1 impairs macrophage-mediated efferocytosis through the inhibition of Rab43 expression. We also determined that the role of Rab43 in efferocytosis is to regulate the cell surface expression of CD91, which is a key receptor for macrophage efferocytosis. More specifically, Rab43 controls the anterograde trafficking of CD91 from the ER to the cell surface. Knocking out *Rab43* arrested the transport of CD91 in the ER and decreased the level of CD91 on the cell surface. Further analysis demonstrated that *Rab43* deletion delayed the inflammation resolution and exacerbated the lung tissue injury in mice with ALI. Therefore, Rab43 is a potentially novel therapeutic target for the treatment of ALI and ARDS.

Extensive evidence demonstrates that impaired efferocytosis serves as a trigger for inflammatory storms and delayed inflammation resolution in ALI/ARDS (37). Mechanistically, restoring normal efferocytosis might limit tissue inflammation and injury (38). Accumulating evidence further shows that damage‐associated molecular pattern molecules (such as HMGB1) are generated from the damaged lung tissue and then released into the BALF, resulting in suppressed efferocytosis (10, 39, 40). Consistent with these findings, our study showed that molecules in the BALF of the ALI/ARDS mouse model also appeared to suppress macrophage-mediated efferocytosis. Specifically, a high concentration of HMGB1 was detected in the BALF. However, in this study, the BALF was obtained from an LPS-induced ALI/ARDS model; thus, we speculated that it was possible that some residual LPS were retained in the BALF. Previous studies have shown that LPS may suppress the macrophage phagocytosis of apoptotic neutrophils *in vitro* (41, 42). For instance, Miao et al. (43) found that continuous LPS stimulation impaired zymosan phagocytosis by peritoneal macrophages *in vivo.* These data indicate that many factors (such as HMGB1 and LPS) within the BALF might directly impact the ability of macrophages to perform phagocytosis on apoptotic cells in the ALI/ARDS model. To specifically elucidate the role of HMGB1 on this process, we blocked its expression using anti-HMGB1 neutralizing antibodies, which significantly attenuated the suppression of efferocytosis previously observed in the BALF. Taken together, these data indicate that extracellular HMGB1 is a major mediator in the BALF impacting the macrophage phagocytosis of apoptotic cells in the ALI/ARDS model.

Furthermore, extracellular HMGB1 can inhibit the macrophage-mediated efferocytosis through multiple mechanisms. For example, HMGB1 can block the phosphatidylserine on neutrophils (10) and the phagocytic receptor αvβ3 on phagocytes (44). Interestingly, we discovered that HMGB1 inhibited the CD91 expression on the cell surface of BMDMs. CD91 is a type I transmembrane receptor that mediates the endocytosis of different ligands (45). The calreticulin-regulated activation of CD91 stimulates Rac-1 and drives the engulfment of ACs (17). Toll-like receptor crosstalk activates CD91 to recruit Rab8a and PI3Kγ for the suppression of inflammatory responses (46). In the present study, cell surface CD91 was blocked using anti-CD91 antibodies, which led to the suppression of efferocytosis by BMDMs. These findings indicate that CD91 plays a key role in efferocytosis. Moreover, we found that the transport of CD91 was arrested in the ER of HMGB1-treated BMDMs. Therefore, we speculate that HMGB1 inhibited the transport of CD91 to the cell surface, thereby impairing BMDM-mediated efferocytosis.

Several Rab GTPases are required for the anterograde transport of key receptors from the intracellular compartments to the cell surface, while the regulation of receptor expression mediates normal physiological function (47). Our previous study revealed that Rab1 controls trafficking of β-adrenergic receptor (β-AR) from the ER to the Golgi body and cell surface, thereby regulating the permeability of endothelial cells (48). In addition, Li et al. (26) reported that Rab43 mediates the transport of non-GPCR membrane proteins from the ER to the cell surface. Furthermore, Jiang et al. (24) showed that Rab11a elevates the level of a disintegrin and metalloproteinase (ADAM) 17 and reduces the CD36 expression level on the cell surface, consequently inhibiting macrophage-mediated efferocytosis. In this study, we found the HMGB1 treatment suppressed the function of Rab43. In turn, the functional loss of Rab43 in myeloid-specific *Rab43* knockout mice significantly repressed the efferocytosis by macrophages, resulting in the delayed inflammation resolution and exacerbated LPS-induced lung tissue injury in the ALI mouse model. Additionally, we found that extracellular HMGB1 downregulated the expression of Rab11a, which negatively affects efferocytosis. These data suggested that Rab43 is a major target of HMGB1; therefore, the effect of HMGB1 on the expression of Rab11 requires further investigation.

Taken together, our results suggest the HMGB1 potentially suppresses the Rab43-regulated transport of CD91 to the cell surface, thereby impairing the macrophage-mediated engulfment of apoptotic cells. Interestingly, other Rab GTPases such as Rab7 (34), Rab20 (49), and Rab35 (50) also positively regulate macrophage-mediated phagocytosis during phagosome formation and maturation. However, further research is required to determine the exact function of Rab43 and the underlying mechanism involved during macrophage-mediated phagocytosis.

In conclusion, our findings reveal that HMGB1 treatment can inhibit BMDM-mediated efferocytosis and delay inflammation resolution by suppressing the Rab43-controlled anterograde transport of CD91 from the ER to the cell surface. Furthermore, we hypothesize that the restoration of Rab43 levels in macrophages to enhance efferocytosis may be a potential therapeutic approach for the treatment of ALI and ARDS.

## Materials and Methods

### Reagents and Antibodies

The reagents used for the study were as follows: rHMGB1 (Sino Biological, Beijing, China; #50913-M01H), CFDA SE Cell Proliferation Assay and Tracking Kit (Beyotime Biotechnology, Shanghai, China; #C0051), Gibco^®^ Cell Dissociation Buffer (Thermo Fisher Scientific, Waltham, MA, USA; #13150016), LPS from *Escherichia coli* 055:B5 (Sigma-Aldrich, St. Louis, MO, USA; #L2880), recombinant macrophage colony-stimulating factor (M-CSF; Bioworld Technology, Bloomington, MN, USA; BK0128), Macrophage Medium, MAM (ScienCell, Carlsbad, CA, USA; #1921), Dil Stain (Thermo Fisher Scientific, #D3911), Pierce™ BCA Protein Assay Kit (Thermo Fisher Scientific, #23225), M-PER™ Protein Extraction Reagent (Thermo Fisher Scientific, #78510), cOmplete™ EDTA-free Protease Inhibitor Cocktail (Sigma-Aldrich, #04693159001), PageRuler™ Prestained Protein Ladder (Thermo Fisher Scientific, #26616), Immobilon Western Chemiluminescent HRP Substrate (Millipore, Temecula, CA, USA; #WBKLS0500), TRIzol™ Reagent (Sigma-Aldrich, #T9424), GoScript™ Reverse Transcription System (Promega, Madison, WI, USA; #A2800), GoTaq qPCR Master Mix (Promega, #A6001), Immunofluorescence Application Solutions Kit (CST, Danvers, MA, USA; #12727), Antifade Mounting Medium with DAPI (Coolaber, Beijing, China; #SL 1841), Cytofix/Cytoperm™ Fixation/Permeabilization Kit (BD Biosciences, San Jose, CA, USA; #554714), Golgi Staining Kit—Green Fluorescence | Cytopainter (Abcam, Cambridge, MA, USA; #ab139483), Protein A/G PLUS-Agarose (Santa Cruz Biotechnology, Santa Cruz, CA, USA; #sc-2003), Enzyme-Linked Immunosorbent Assay (ELISA) Kit For High Mobility Group Protein 1 (Cloud-Clone Corp., Wuhan, China; #SEA399Mu), FuGENE^®^ HD Transfection Reagent (Promega, #2311), MS columns (Miltenyi Biotec, Bergisch Gladbach, Germany; #5190110006), Dexamethasone (MCE, Monmouth Junction, NJ, USA; #HY-14648), PE Annexin V Apoptosis Detection Kit I (BD biosciences, #559763), and Mem-PERTM Plus Membrane Protein Extraction Kit (Thermo Fisher Scientific, #89842).

The antibodies used include the PE anti-mouse CD91 Antibody (Bioss, Beijing, China; #bs-10920R-PE), APC anti-mouse F4/80 Antibody (BioLegend, #123116), FITC anti-mouse F4/80 Antibody (BioLegend, #123108), APC/Cyanine7 anti-mouse F4/80 Antibody (BioLegend, #123118), PE/Cyanine7 anti-mouse Ly-6G/Ly-6C (Gr-1) Antibody (BioLegend, #108416), anti-CD16/32 Antibody (BioLegend, #101302), APC anti-mouse CD11c Antibody (BioLegend, #117310), PerCP labeled Anti-Mouse/Human CD11b Antibody (Elabscience, Wuhan, China; #E-AB-F1081F), Anti-RAB43 Antibody (Santa Cruz Biotechnology, #sc-515460), Anti-β-Actin Antibody (CST, #4970S), Anti-CD91 Antibody (Abcam, #ab92544), Calnexin Antibody (Thermo Fisher Scientific, AF18, #MA3-027), Alexa Fluor^®^ 488-conjugated AffiniPure Goat Anti-Mouse IgG (Jackson Immunoresearch, West Grove, PA, USA; #115-545-003), Alexa Fluor^®^ 594-conjugated AffiniPure Goat Anti-Rabbit IgG (Jackson Immunoresearch, #111-585-003), Anti-F4/80 Micro-Beads UltraPure, mouse (Miltenyi Biotec, #130-110-443), Anti-FLAG Antibody (CST, #14793), Anti-GFP Antibody (CST, #2956), Mouse Anti-rabbit IgG (Conformation Specific) (L27A9) mAb (HRP Conjugate) (CST, #5127), anti-HMGB1 Neutralizing antibody (Arigo, #ARG66714), ATP1A1 Polyclonal Antibody (Proteintech, #14418-1-AP), BV421 anti-mouse MerTK Antibody (BD Biosciences, #747837), and PE anti-mouse CD36 Antibody (BioLegend, #102605).

### Animals and ALI/ARDS Model Construction

All animal experiments were carried out following the guidelines of the Animal Care and Use Committee of the Third Military Medical University and were approved by the local Administration District Official Committee of Third Military Medical University, Chongqing, China. Rab43-cKO mice were generated as described in a previous study (30). In brief, we generated the Rab43-cKO mice by crossing *LysM-Cre* ^+/+^ mice with *Rab43^floxp/floxp^* mice; *Rab43^floxp/floxp^/LysMCre^−/−^* mice were used as the *Rab43* wild-type strain (Rab43-C) for comparison. For construction of the ALI/ARDS model, 5 mg/kg LPS (*E. coli* 055:B5) was directly instilled into the tracheas of 6- to 8-week-old mice lightly sedated with isoflurane using a modified feeding needle. The mice were sacrificed at indicated time points after LPS administration. The lung tissues were excised and subjected to hematoxylin and eosin (H&E) staining for histological evaluation.

### Isolation and Culture of Macrophages

The BMDMs were harvested following the methods of a previous study (51). Briefly, 6- to 8-week-old *Rab43 loxp^+/+^ Cre^−/−^* or *Rab43 loxp^+/+^ Cre^+/+^* mice were sacrificed. The bone marrow cells isolated from the tibia and femur were cultured in MAM containing 10% fetal bovine serum and 100 ng/ml M-CSF for 6 days.

### RNA Isolation and Real-Time qPCR

TRIzol™ and GoScript™ Reverse Transcription System reagents were used to extract and reverse-transcribe (2 μg) total RNA into cDNA. The cDNA sample and GoTaq qPCR Master Mix were used in qPCR to measure the relative expression of target genes. The expression values were normalized using β-actin as internal control. Relative mRNA expression was quantitated with the 2−△△Ct method. The qPCR primers were: *Rab43* (forward: 5′-GGTCAAGTTACAGATTTGGGAC-3′, reverse: 5′-GTACTTCCTCACATCCTCGATC-3′); *Rab11a* (forward: 5′-AGTGATTTACGTCATCTCAGGG-3′, reverse: 5′-GTTGCTTGGAGACATGTCATTT-3′) *Rab7* (forward: 5′-AAAGACCTCTCTCATGAACCAG-3′, reverse: 5′- -CAGTCACATCAAACACCAGAAC-3′); and β-actin (forward: 5′-CTACCTCATGAAGATCCTGACC-3′, reverse: 5′-CACAGCTTCTCTTTGATGTCAC-3′).

### Plasmid Transfection

The pcDNA3.1-3xflag-Rab43 and pcDNA3.1-GFP-Rab43 plasmids were purchased from Sangon Biotech (Shanghai, China). The RAW264.7 cells were transfected with the plasmids using FuGENE HD Transfection Reagent, according to the protocol of the manufacturer.

### BALF Sample Collection

The lungs were infused four times with 500 μl cold sterile PBS (total volume of 2 ml) to collect the BALF at indicated time points after LPS or PBS treatment. The BALF samples were centrifuged at 3,000×*g* for 10 min at 4°C. The cell-free supernatants were filtered using a 0.45-μm polyvinylidene fluoride membrane. The filtrate was temporarily stored at –80°C and then analyzed to determine the cytokine and protein concentrations.

### Flow Cytometry Analysis of Cell Surface CD91

BMDMs were incubated with the PE anti-CD91 antibody (1:100) for 30 min on ice, washed twice, and subjected to flow cytometry. For the *in vivo* evaluation, cells derived from alveolar lavage fluid of ARDS mice were incubated with PE anti-CD91 antibody (1:100), APC/Cyanine7 F4/80 antibody (1:100), PerCP CD11b antibody (1:100), and APC CD11c antibody (1:100) for 30 min on ice, washed twice, and subjected to flow cytometry. The mean fluorescence intensity of CD91 was assessed in F4/80^+^CD11c^+^CD11b^−^ cells. Flow cytometry analysis was performed using FACSCanto™ II Clinical Flow Cytometry System (BD Biosciences). The flow cytometric data were collected using a FACScan cytometer CANTO II (Becton Dickinson, Franklin Lakes, NJ, USA) and analyzed using FlowJo software (TreeStar, Ashland, OR, USA).

### Immunofluorescence Staining

The BMDMs or RAW264.7 cells were grown in glass-bottom dishes, fixed with 4% paraformaldehyde, and stained using Immunofluorescence Application Solutions Kit (CST, #12727), following the instructions of the manufacturer. The cells were observed using a Leica confocal microscope (Leica Microsystems, Wetzlar, Germany) and analyzed using ImageJ software (National Institutes of Health, Bethesda, MD, USA; http://rsb.info.nih.gov/nih-image/) or Leica Application Suite (LAS) AF version 2.3.0 software (Leica Microsystems).

### *In Vitro* Phagocytosis Assay

The apoptotic thymocytes were harvested after the thymocytes were treated with 1 mM dexamethasone (MCE, #HY-14648) at 37°C for 6 h. The macrophage-mediated efferocytosis *in vitro* was evaluated using immunofluorescence staining and flow cytometry. For immunofluorescence staining, the Rab43-C or Rab43-cKO macrophages were seeded in cell culture dishes (NEST Biotechnology, Wuxi, China; #801002) overnight. The macrophages were incubated with apoptotic thymocytes labeled with carboxyfluorescein succinimidyl ester (CFSE; 10mM, 1:1,000) at a 1:10 ratio and then cultured in PBS at 37°C for 120 min. The macrophages were subsequently stained with Dil Stain (1:1,000). The cells were viewed using a Leica confocal microscope (Leica Microsystems).

For flow cytometry, the BMDMs were incubated with CFSE-labeled apoptotic thymocytes at a 1:10 ratio and then cultured in PBS at 37°C for 120 min. The macrophages were labeled with the anti-F4/80 antibody (1:200). The flow cytometric data were collected using a FACScan cytometer CANTO II (Becton Dickinson, Franklin Lakes, NJ, USA) and analyzed using FlowJo software for Windows (Tree Star).

### *In Vivo* Phagocytosis Assay

To investigate the macrophage-mediated phagocytosis *in vivo* (52), the cells from the BALF sample were labeled with anti-F4/80 and Ly-6G antibodies (1:200) for 20 min, and then permeabilized using Intracellular Fixation & Permeabilization Buffer (BD Biosciences, #554714). Subsequently, the permeabilized cells were incubated with anti-Ly-6G antibody (1:200). The ratio of F4/80^+^Ly6G^+^ cells over the F4/80^+^ cells was detected by flow cytometry analysis.

### Co-Localization of Endogenous CD91 With the Golgi Body

The BMDMs were fixed with 4% paraformaldehyde at 37°C for 10 min. A sufficient volume of Dual Detection Reagent (Abcam, #ab139483) was dispensed to cover the monolayer cells (1:100 dilution). The samples were protected from light and incubated at 4°C for 30 min. After blocking for 1 h, the cultured Rab43-C and Rab43-cKO BMDMs were stained with anti-CD91 antibody (1:100 dilution) and then with Alexa Fluor^®^ 594-conjugated secondary antibodies (1:200 dilution).

### Coimmunoprecipitation and Western Blot Analysis

The pcDNA3.1-3xflag-Rab43 plasmid was transfected into RAW264.7 cells. After 48 h, total proteins were extracted from the cells using M-PER™ Protein Extraction Reagent and centrifuged at 14,000×*g* for 20 min at 4°C. The supernatant was incubated with anti-FLAG antibody (1:50) overnight and then with protein A/G agarose beads for 3 h at 4°C. Finally, western blotting was performed to detect the expression of CD91.

### Apoptotic Neutrophil Counts

BALF from ARDS mice was detected using PE Annexin V Apoptosis Detection Kit I as described previously (52). Annexin V+/Ly6G+ cells were counted as apoptotic neutrophils.

### HMGB1 Neutralization

The neutralization assay was performed as described previously (15). In brief, BALF from ARDS mice was preincubated with anti-HMGB1 neutralizing antibody for 2 h; isotype IgM was used as control.

### W/D Lung Weight Ratio Measurement

After LPS treatment, the mice were sacrificed, and the lungs were excised and immediately weighed to obtain the wet weight. The lungs were subsequently dried in an oven at 90°C for 72 h and then weighed to determine the dry weight.

### Lung Histological Analysis and Lung Injury Scoring

The mice were sacrificed at the indicated times after LPS administration. The lungs were obtained, fixed with 4% paraformaldehyde, embedded in paraffin, and stained with H&E. The severity of lung injury was evaluated by three blinded investigators, following a previously reported histological semiquantitative scoring system (53). Lung fields were scored per slide at ×20 magnification.

### Statistical Analysis

The data from replicate experiments are presented as means ± SD. All experiments were conducted at least three times. The difference between two groups was analyzed using Student’s *t*-test, whereas differences among three or more groups were evaluated using one-way analysis of variance. Differences with *P*-values less than 0.05 were considered statistically significant.

## Data Availability Statement

The raw data supporting the conclusions of this article will be made available by the authors, without undue reservation.

## Ethics Statement

The animal study was reviewed and approved by the local Administration District Official Committee of Third Military Medical University, Chongqing, China.

## Author Contributions

GW and BH made substantial contributions to the conception and design of the study. YW, WZ, YX, ZG, DW, JZ, and HQ conducted the experiments. BH, YW, and WZ analyzed the experimental data. BH, YW, and WZ wrote the manuscript. HQ, BH and GW edited and revised the manuscript. All authors listed have made a substantial, direct, and intellectual contribution to the work and approved it for publication.

## Funding

This work was supported by the National Natural Science Foundation of China (Nos. 81873422 and 81873421) and the Natural Science Foundation of Chongqing (Nos. cstc2019jcyj-msxmS0367 and cstc2021ycjh-bgzxm0011).

## Conflict of Interest

The authors declare that the research was conducted in the absence of any commercial or financial relationships that could be construed as a potential conflict of interest.

## Publisher’s Note

All claims expressed in this article are solely those of the authors and do not necessarily represent those of their affiliated organizations, or those of the publisher, the editors and the reviewers. Any product that may be evaluated in this article, or claim that may be made by its manufacturer, is not guaranteed or endorsed by the publisher.

## Abbreviations

Rab43-C, Rab43^floxp/floxp^/LysMCre^−/−^; Rab43-cKO, *Rab43^floxp/floxp^/LysMCre^+/+^*; rHMGB1, recombinant high-mobility group box 1 protein; ALI, acute lung injury; ARDS, acute respiratory distress syndrome; BALF, bronchoalveolar lavage fluid; BMDM, bone marrow-derived macrophage; CFSE, carboxy fluorescein succinimidyl ester; CLSM, confocal laser-scanning microscopy; Dex, dexamethasone; ER, endoplasmic reticulum; GFP, green fluorescent protein; LPS, lipopolysaccharide; MFI, mean fluorescence intensity; PI, phagocytic index.

## References

[1] Thompson BT Chambers RC Liu KD. Acute Respiratory Distress Syndrome. N Engl J Med (2017) 377(6):562–72. doi: 10.1056/NEJMra1608077 28792873

[2] Fan E Brodie D Slutsky AS. Acute Respiratory Distress Syndrome: Advances in Diagnosis and Treatment. Jama (2018) 319(7):698–710. doi: 10.1001/jama.2017.21907 29466596

[3] Puneet P Moochhala S Bhatia M. Chemokines in Acute Respiratory Distress Syndrome. Am J Physiol Lung Cell Mol Physiol (2005) 288(1):L3–15. doi: 10.1152/ajplung.00405.2003 15591040 PMC7191630

[4] Zhang K Jin Y Lai D Wang J Wang Y Wu X. RAGE-Induced ILC2 Expansion in Acute Lung Injury Due to Haemorrhagic Shock. Thorax (2020) 75(3):209–19. doi: 10.1136/thoraxjnl-2019-213613 PMC7063398 31937554

[5] Kang R Chen R Zhang Q Hou W Wu S Cao L. HMGB1 in Health and Disease. Mol Aspects Med (2014) 40:1–116. doi: 10.1016/j.mam.2014.05.001 25010388 PMC4254084

[6] Kwak MS Kim HS Lee B Kim YH Son M Shin JS. Immunological Significance of HMGB1 Post-Translational Modification and Redox Biology. Front Immunol (2020) 11:1189. doi: 10.3389/fimmu.2020.01189 32587593 PMC7297982

[7] Wang H Bloom O Zhang M Vishnubhakat JM Ombrellino M Che J. HMG-1 as a Late Mediator of Endotoxin Lethality in Mice. Science (1999) 285(5425):248–51. doi: 10.1126/science.285.5425.248 10398600

[8] Wang H Ward MF Sama AE. Targeting HMGB1 in the Treatment of Sepsis. Expert Opin Ther Targets (2014) 18(3):257–68. doi: 10.1517/14728222.2014.863876 PMC3945414 24392842

[9] Davis K Banerjee S Friggeri A Bell C Abraham E Zerfaoui M. Poly(ADP-Ribosyl)Ation of High Mobility Group Box 1 (HMGB1) Protein Enhances Inhibition of Efferocytosis. Mol Med (2012) 18(1):359–69. doi: 10.2119/molmed.2011.00203 PMC3356430 22204001

[10] Liu G Wang J Park YJ Tsuruta Y Lorne EF Zhao X. High Mobility Group Protein-1 Inhibits Phagocytosis of Apoptotic Neutrophils Through Binding to Phosphatidylserine. J Immunol (2008) 181(6):4240–6. doi: 10.4049/jimmunol.181.6.4240 PMC2597447 18768881

[11] Savill J Fadok V. Corpse Clearance Defines the Meaning of Cell Death. Nature (2000) 407(6805):784–8. doi: 10.1038/35037722 11048729

[12] Cheng P Li S Chen H. Macrophages in Lung Injury, Repair, and Fibrosis. Cells (2021) 10(2):436. doi: 10.3390/cells10020436 33670759 PMC7923175

[13] Grailer JJ Haggadone MD Sarma JV Zetoune FS Ward PA. Induction of M2 Regulatory Macrophages Through the β2-Adrenergic Receptor With Protection During Endotoxemia and Acute Lung Injury. J Innate Immun (2014) 6(5):607–18. doi: 10.1159/000358524 PMC4159611 24642449

[14] Kimura H Suzuki M Konno S Shindou H Shimizu T Nagase T. Orchestrating Role of Apoptosis Inhibitor of Macrophage in the Resolution of Acute Lung Injury. J Immunol (2017) 199(11):3870–82. doi: 10.4049/jimmunol.1601798 29070674

[15] Grégoire M Uhel F Lesouhaitier M Gacouin A Guirriec M Mourcin F. Impaired Efferocytosis and Neutrophil Extracellular Trap Clearance by Macrophages in ARDS. Eur Respir J (2018) 52(2):1702590. doi: 10.1183/13993003.02590-2017 29946009

[16] Ogden CA deCathelineau A Hoffmann PR Bratton D Ghebrehiwet B Fadok VA. C1q and Mannose Binding Lectin Engagement of Cell Surface Calreticulin and CD91 Initiates Macropinocytosis and Uptake of Apoptotic Cells. J Exp Med (2001) 194(6):781–95. doi: 10.1084/jem.194.6.781 PMC2195958 11560994

[17] Gardai SJ McPhillips KA Frasch SC Janssen WJ Starefeldt A Murphy-Ullrich JE. Cell-Surface Calreticulin Initiates Clearance of Viable or Apoptotic Cells Through Trans-Activation of LRP on the Phagocyte. Cell (2005) 123(2):321–34. doi: 10.1016/j.cell.2005.08.032 16239148

[18] Mueller PA Kojima Y Huynh KT Maldonado RA Ye J Tavori H. Macrophage LRP1 (Low-Density Lipoprotein Receptor-Related Protein 1) Is Required for the Effect of CD47 Blockade on Efferocytosis and Atherogenesis. Arterioscler Thromb Vasc Biol (2022) 42(1):e1–9. doi: 10.1161/ATVBAHA.121.316854 PMC8702482 34758632

[19] Yancey PG Ding Y Fan D Blakemore JL Zhang Y Ding L. Low-Density Lipoprotein Receptor-Related Protein 1 Prevents Early Atherosclerosis by Limiting Lesional Apoptosis and Inflammatory Ly-6Chigh Monocytosis: Evidence That the Effects Are Not Apolipoprotein E Dependent. Circulation (2011) 124(4):454–64. doi: 10.1161/CIRCULATIONAHA.111.032268 PMC3144781 21730304

[20] Wujak L Schnieder J Schaefer L Wygrecka M. LRP1: A Chameleon Receptor of Lung Inflammation and Repair. Matrix Biol (2018) 68-69:366–81. doi: 10.1016/j.matbio.2017.12.007 29262309

[21] Mantuano E Brifault C Lam MS Azmoon P Gilder AS Gonias SL. LDL Receptor-Related Protein-1 Regulates Nfκb and microRNA-155 in Macrophages to Control the Inflammatory Response. Proc Natl Acad Sci USA (2016) 113(5):1369–74. doi: 10.1073/pnas.1515480113 PMC4747752 26787872

[22] Yancey PG Blakemore J Ding L Fan D Overton CD Zhang Y. Macrophage LRP-1 Controls Plaque Cellularity by Regulating Efferocytosis and Akt Activation. Arterioscler Thromb Vasc Biol (2010) 30(4):787–95. doi: 10.1161/ATVBAHA.109.202051 PMC2845445 20150557

[23] Dong C Filipeanu CM Duvernay MT Wu G. Regulation of G Protein-Coupled Receptor Export Trafficking. Biochim Biophys Acta (2007) 1768(4):853–70. doi: 10.1016/j.bbamem.2006.09.008 PMC1885203 17074298

[24] Jiang C Liu Z Hu R Bo L Minshall RD Malik AB. Inactivation of Rab11a GTPase in Macrophages Facilitates Phagocytosis of Apoptotic Neutrophils. J Immunol (2017) 198(4):1660–72. doi: 10.4049/jimmunol.1601495 PMC5296368 28053235

[25] Taefehshokr N Yin C Heit B. Rab GTPases in the Differential Processing of Phagocytosed Pathogens Versus Efferocytosed Apoptotic Cells. Histol Histopathol (2021) 36(2):123–35.doi: 10.14670/HH-18-252 32990320

[26] Li C Wei Z Fan Y Huang W Su Y Li H. The GTPase Rab43 Controls the Anterograde ER-Golgi Trafficking and Sorting of GPCRs. Cell Rep (2017) 21(4):1089–101. doi: 10.1016/j.celrep.2017.10.011 PMC6051424 29069590

[27] Steger M Diez F Dhekne HS Lis P Nirujogi RS Karayel O. Systematic Proteomic Analysis of LRRK2-Mediated Rab GTPase Phosphorylation Establishes a Connection to Ciliogenesis. Elife (2017) 6:e31012. doi: 10.7554/eLife.31012 29125462 PMC5695910

[28] Inoue M Ishibashi Y Nogawa H Yasue T. Carbocisteine Promotes Phagocytosis of Apoptotic Cells by Alveolar Macrophages. Eur J Pharmacol (2012) 677(1-3):173–9. doi: 10.1016/j.ejphar.2011.12.026 22222820

[29] Seto S Tsujimura K Koide Y. Rab GTPases Regulating Phagosome Maturation Are Differentially Recruited to Mycobacterial Phagosomes. Traffic (2011) 12(4):407–20. doi: 10.1111/j.1600-0854.2011.01165.x 21255211

[30] Luo B Wang J Liu Z Shen Z Shi R Liu YQ. Phagocyte Respiratory Burst Activates Macrophage Erythropoietin Signalling to Promote Acute Inflammation Resolution. Nat Commun (2016) 7:12177. doi: 10.1038/ncomms12177 27397585 PMC4942576

[31] Friggeri A Yang Y Banerjee S Park YJ Liu G Abraham E. HMGB1 Inhibits Macrophage Activity in Efferocytosis Through Binding to the Alphavbeta3-Integrin. Am J Physiol Cell Physiol (2010) 299(6):C1267–76. doi: 10.1152/ajpcell.00152.2010 PMC3006331 20826760

[32] Wang X Bu HF Zhong W Asai A Zhou Z Tan XD. MFG-E8 and HMGB1 Are Involved in the Mechanism Underlying Alcohol-Induced Impairment of Macrophage Efferocytosis. Mol Med (2013) 19(1):170–82. doi: 10.2119/molmed.2012.00260 PMC3745596 23552724

[33] Zhou M Fang H Du M Li C Tang R Liu H. The Modulation of Regulatory T Cells via HMGB1/PTEN/β-Catenin Axis in LPS Induced Acute Lung Injury. Front Immunol (2019) 10:1612. doi: 10.3389/fimmu.2019.01612 31402909 PMC6669370

[34] Aflaki E Borger DK Grey RJ Kirby M Anderson S Lopez G. Efferocytosis Is Impaired in Gaucher Macrophages. Haematologica (2017) 102(4):656–65. doi: 10.3324/haematol.2016.155093 PMC5395106 28011901

[35] Arandjelovic S Ravichandran KS. Phagocytosis of Apoptotic Cells in Homeostasis. Nat Immunol (2015) 16(9):907–17. doi: 10.1038/ni.3253 PMC4826466 26287597

[36] Mueller PA Zhu L Tavori H Huynh K Giunzioni I Stafford JM. Deletion of Macrophage Low-Density Lipoprotein Receptor-Related Protein 1 (LRP1) Accelerates Atherosclerosis Regression and Increases C-C Chemokine Receptor Type 7 (CCR7) Expression in Plaque Macrophages. Circulation (2018) 138(17):1850–63. doi: 10.1161/CIRCULATIONAHA.117.031702 PMC6343494 29794082

[37] Hiruma T Tsuyuzaki H Uchida K Trapnell BC Yamamura Y Kusakabe Y. IFN-β Improves Sepsis-Related Alveolar Macrophage Dysfunction and Postseptic Acute Respiratory Distress Syndrome-Related Mortality. Am J Respir Cell Mol Biol (2018) 59(1):45–55. doi: 10.1165/rcmb.2017-0261OC 29365277 PMC6835072

[38] Elliott MR Koster KM Murphy PS. Efferocytosis Signaling in the Regulation of Macrophage Inflammatory Responses. J Immunol (2017) 198(4):1387–94. doi: 10.4049/jimmunol.1601520 PMC5301545 28167649

[39] Han S Mallampalli RK. The Acute Respiratory Distress Syndrome: From Mechanism to Translation. J Immunol (2015) 194(3):855–60. doi: 10.4049/jimmunol.1402513 PMC4299926 25596299

[40] Schaper F de Leeuw K Horst G Bootsma H Limburg PC Heeringa P. High Mobility Group Box 1 Skews Macrophage Polarization and Negatively Influences Phagocytosis of Apoptotic Cells. Rheumatol (Oxford) (2016) 55(12):2260–70. doi: 10.1093/rheumatology/kew324 27632996

[41] Feng X Deng T Zhang Y Su S Wei C Han D. Lipopolysaccharide Inhibits Macrophage Phagocytosis of Apoptotic Neutrophils by Regulating the Production of Tumour Necrosis Factor α and Growth Arrest-Specific Gene 6. Immunology (2011) 132(2):287–95. doi: 10.1111/j.1365-2567.2010.03364.x PMC3050451 21039473

[42] Michlewska S Dransfield I Megson IL Rossi AG. Macrophage Phagocytosis of Apoptotic Neutrophils Is Critically Regulated by the Opposing Actions of Pro-Inflammatory and Anti-Inflammatory Agents: Key Role for TNF-Alpha. FASEB J (2009) 23(3):844–54. doi: 10.1096/fj.08-121228 18971259

[43] Miao J Ye P Lan J Ye S Zhong J Gresham A. Paeonol Promotes the Phagocytic Ability of Macrophages Through Confining HMGB1 to the Nucleus. Int Immunopharmacol (2020) 89(Pt B):107068. doi: 10.1016/j.intimp.2020.107068 33091813

[44] Jun JI Kim KH Lau LF. The Matricellular Protein CCN1 Mediates Neutrophil Efferocytosis in Cutaneous Wound Healing. Nat Commun (2015) 6:7386. doi: 10.1038/ncomms8386 26077348 PMC4480344

[45] Lillis AP Van Duyn LB Murphy-Ullrich JE Strickland DK. LDL Receptor-Related Protein 1: Unique Tissue-Specific Functions Revealed by Selective Gene Knockout Studies. Physiol Rev (2008) 88(3):887–918. doi: 10.1152/physrev.00033.2007 18626063 PMC2744109

[46] Luo L Wall AA Tong SJ Hung Y Xiao Z Tarique AA. TLR Crosstalk Activates LRP1 to Recruit Rab8a and PI3Kγ for Suppression of Inflammatory Responses. Cell Rep (2018) 24(11):3033–44. doi: 10.1016/j.celrep.2018.08.028 30208326

[47] Wang G Wu G. Small GTPase Regulation of GPCR Anterograde Trafficking. Trends Pharmacol Sci (2012) 33(1):28–34. doi: 10.1016/j.tips.2011.09.002 22015208 PMC3259232

[48] Li Y Wang G Lin K Yin H Zhou C Liu T. Rab1 GTPase Promotes Expression of Beta-Adrenergic Receptors in Rat Pulmonary Microvascular Endothelial Cells. Int J Biochem Cell Biol (2010) 42(7):1201–9. doi: 10.1016/j.biocel.2010.04.009 PMC4792279 20417717

[49] Egami Y Araki N. Rab20 Regulates Phagosome Maturation in RAW264 Macrophages During Fc Gamma Receptor-Mediated Phagocytosis. PloS One (2012) 7(4):e35663. doi: 10.1371/journal.pone.0035663 22545127 PMC3335809

[50] Egami Y Fukuda M Araki N. Rab35 Regulates Phagosome Formation Through Recruitment of ACAP2 in Macrophages During Fcγr-Mediated Phagocytosis. J Cell Sci (2011) 124(Pt 21):3557–67. doi: 10.1242/jcs.083881 22045739

[51] Luo B Gan W Liu Z Shen Z Wang J Shi R. Erythropoeitin Signaling in Macrophages Promotes Dying Cell Clearance and Immune Tolerance. Immunity (2016) 44(2):287–302. doi: 10.1016/j.immuni.2016.01.002 26872696

[52] Schwab JM Chiang N Arita M Serhan CN. Resolvin E1 and Protectin D1 Activate Inflammation-Resolution Programmes. Nature (2007) 447(7146):869–74. doi: 10.1038/nature05877 PMC2757086 17568749

[53] D'Alessio FR Tsushima K Aggarwal NR Mock JR Eto Y Garibaldi BT. Resolution of Experimental Lung Injury by Monocyte-Derived Inducible Nitric Oxide Synthase. J Immunol (2012) 189(5):2234–45. doi: 10.4049/jimmunol.1102606 PMC3424351 22844117

