# Supplementary material for: Extracellular HMGB1 Impairs Macrophage-Mediated Efferocytosis by Suppressing the Rab43-Controlled Cell Surface Transport of CD91
Source: Front Immunol. 2022 Mar 22;13:767630. doi: 10.3389/fimmu.2022.767630 (PMC8980266; doi:10.3389/fimmu.2022.767630)
Supplement: Supplementary file 1 [file DataSheet_1.pdf]

Supplement Figure 1.

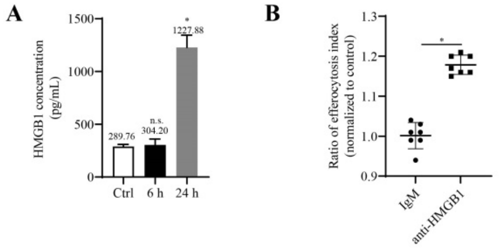

Figure S1. Effects of recombinant HMGB1 (rHMGB1) treatment on the macrophage mediated efferocytosis.

A. The C57BL/6 wild-type (WT) were inject LPS (5mg/kg, i.t.) for 24 hours, HMGB1 levels were measured by ELISA assay in bronchoalveolar lavage fluid (BALF) from PBS-, LPS-treated mice. Mean±SD (n = 4–6 samples/group), *p < 0.05 versus the PBS-treated group. **B**. Bronchoalveolar lavage fluid (BALF) from ARDS mice were preincubated with anti-HMGB1 neutralizing antibody for 2 h before incubation with BMDMs from WT mice for 12 h, isotype igM was used as control. *p < 0.05 versus the BALF-treated group.

Supplement Figure 2.

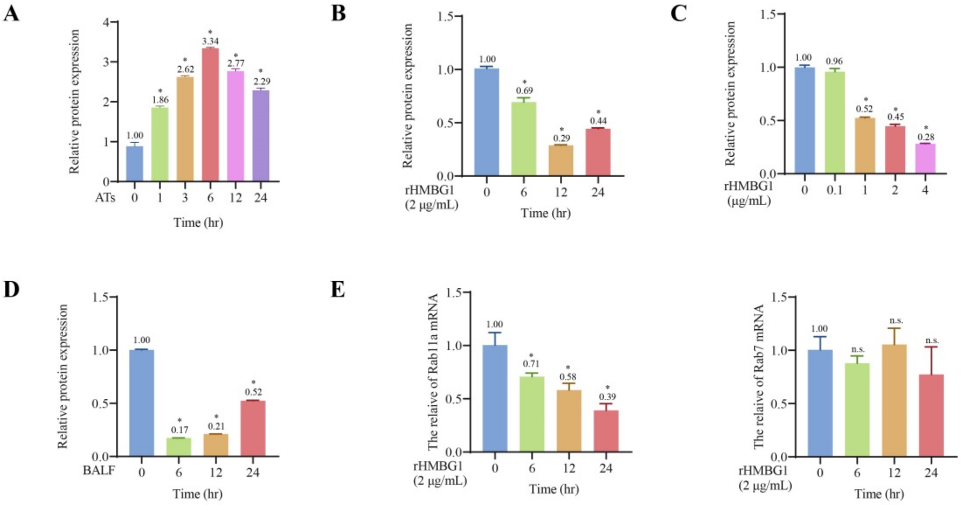

Figure S2. HMGB1 suppressed the expression of Rab43.

A, B, C and D: Relative protein expression for Figure 2A, 2B, 2C, 2D. **E.** qPCR analyses of Rab11a and Rab7 mRNA levels. The BMDMs were treated with 2 μg/mL recombinant HMGB1 (rHMGB1) at indicated time points (0, 6, 12 and 24 h) (C and D). *P < 0.05 versus 0 h.

Supplement Figure 3.

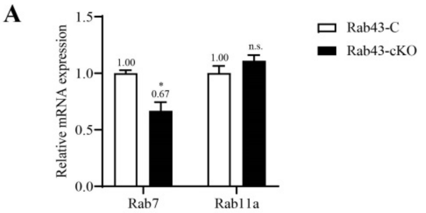

Figure S3. Rab43 deficiency impaired efferocytosis by macrophages.

A: The expression of Rab11a and Rab7 in Rab43-C and Rab43-cKO BMDMs were measured by qPCR. *P < 0.05 versus Rab43-C group. ns, not statistically significant.

Supplement Figure 4.

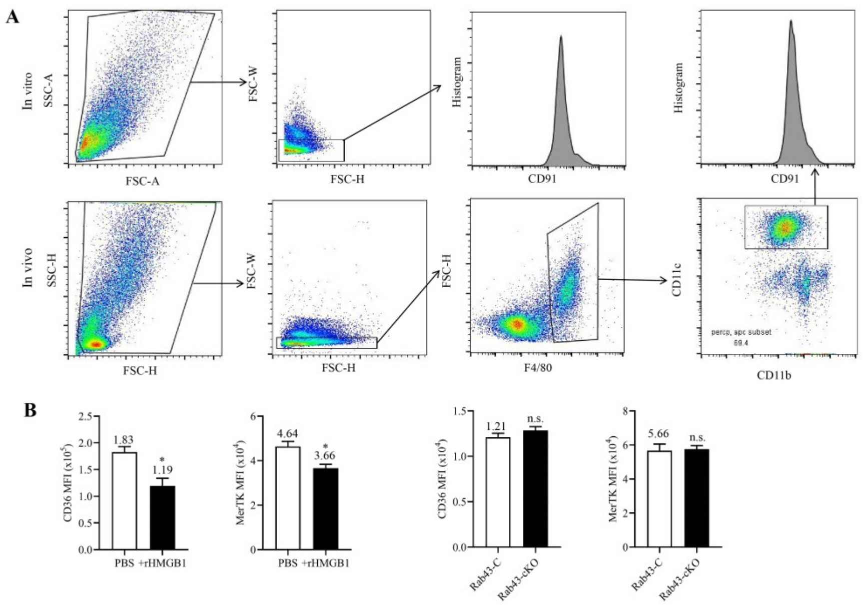

Figure S4. Effects of Rab43 knockout on the cell surface transport of endogenous CD91.

A: Gating strategies for Figure 5A and B. **B**. The BMDMs were treated with 2 μg/mL rHMGB1 for 12 h. The level of CD36 and MerTK on the cell surface was detected using fluorescence-activated cell sorting (FACS). *P < 0.05 versus the PBS treated group. The BMDMs were isolated from Rab43-C and Rab43-cKO mice. The level of CD36 and MerTK on the cell surface was detected using fluorescence-activated cell sorting (FACS). ns, not statistically significant versus Rab43-C group.
